# Chest X-rays and Lung Ultrasound Are Not Interchangeable in Intensive Care Practice

**DOI:** 10.3390/diagnostics14010082

**Published:** 2023-12-29

**Authors:** Stefan Schmidt, Nico Behnke, Jana-Katharina Dieks

**Affiliations:** 1 Department of Anesthesiology, Emergency and Intensive Care Medicine, University Hospital Goettingen, Georg August University, Robert-Koch-Str. 40, 37075 Goettingen, Germany; 2 Department of Pediatric Cardiology and Pediatric Intensive Care Medicine, University Hospital Goettingen, Georg August University, Robert-Koch-Str. 40, 37075 Goettingen, Germany; jana.dieks@med.uni-goettingen.de; 3 Institute for Diagnostic and Interventional Radiology, University Hospital Goettingen, Georg August University, Robert-Koch-Str. 40, 37075 Goettingen, Germany; nico.behnke@med.uni-goettingen.de

**Keywords:** chest ultrasound, point-of-care lung ultrasound, thoracic ultrasound, critical care, chest radiography

## Abstract

Purpose: Data comparing lung ultrasound (LUS) and chest X-rays (CXRs) have increased over the past years. However, there still is a lack of knowledge as to how these modalities compare with one another in the critical care setting, and several factors, including artificial study conditions, limit the generalizability of most published studies. Our study aimed to analyze the performance of LUS in comparison with CXRs in real-world critical care practice. Materials and Methods: This study presents new data from the prospective FASP-ICU trial. A total of 209 corresponding datasets of LUS and CXR results from 111 consecutive surgical ICU patients were subanalyzed, and categorial findings were compared. Statistical analysis was performed on the rates of agreement between the different imaging modalities. Results: A total of 1162 lung abnormalities were detected by LUS in ICU patients compared with 1228 detected by CXR, a non-significant difference (*p* = 0.276; 95% CI −0.886 to 0.254). However, the agreement rates varied between the observed abnormalities: the rate of agreement for the presence of interstitial syndrome ranged from 0 to 15%, consolidation from 0 to 56%, basal atelectasis from 33.9 to 49.34%, pleural effusion from 40.65 to 50%, and compression atelectasis from 14.29 to 19.3%. The rate of agreement was 0% for pneumothorax and 20.95% for hypervolemia. Conclusions: LUS does not detect more lung abnormalities in real-world critical care practice than CXRs, although a higher sensitivity of LUS has been reported in previous studies. Overall, low agreement rates between LUS and CXRs suggest that these diagnostic techniques are not equivalent but instead are complementary and should be used alongside each other.

## 1. Introduction

Lung ultrasound (LUS) is a relatively new technique, is simple to perform in acute care settings, and has a rapid learning curve for the examiner. It is a nonionizing, inexpensive technique that can be performed rapidly. In comparison to other ultrasound modalities, LUS analysis is partially based on the recognition of imaging artifacts, which makes it easily accessible even for non-expert operators. LUS has previously been shown to have a greater sensitivity in comparison to conventional radiologic imaging techniques [1,2,3], particularly in diagnosing pneumothoraces [4], consolidation [5], interstitial syndrome [6,7], cardiogenic pulmonary edema [8], and COVID-19 pneumonia [9] when compared to a chest X-ray (CXR).

Until recently, undertaking daily routine antero-posterior (AP) CXRs was common practice in ICUs. The justification for this was the prompt detection and, consequently, earlier treatment of pathologies detectable by CXR. The practice of undertaking daily routine CXRs on ICUs was largely abandoned after Heyblum et al., in 2009, proved that the strategy of undertaking CXRs only when required was non-inferior to daily routine CXRs [10]. Even before the implementation of LUS as a standard technique in the ICU, a meta-analysis suggested that a restrictive CXR strategy was not associated with harm [11].

In many institutions, the advantage of timely, radiation-free, and readily available LUS examinations in the ICU has further reduced the frequency of CXRs. The utility of LUS in acute settings is supported by a reasonable body of evidence [12]. However, study conditions that may not be applicable to the real world, such as select patient populations, may result in the maximization of pretest probabilities and are just one of many concerns, limitations, and biases surrounding some of these studies comparing LUS and CXRs. Consequently, there are only limited studies that are applicable to daily clinical practice in ICUs.

Previous studies have shown that LUS has a greater sensitivity and similar specificity for detecting lung abnormalities in comparison to a CXR. However, fully assessing the accuracy of LUS compared with CXR across all diagnoses and conditions is difficult; in addition to the above-mentioned maximized pretest probabilities and artificial study conditions, particular conditions and situations, such as post-surgical patients, add to the difficulty of assessing accuracy. Accuracy in specific conditions has been reported; in pneumonia, for example, a systematic review and meta-analysis of 742 patients reported a pooled sensitivity of 95% for LUS and a specificity of 90%, whereas a plain CXR showed a sensitivity of 77% and a specificity of 91% in comparison [13]. In a low-resource setting, a similar sensitivity but lower specificity for LUS has been shown [14]. Further information on sensitivities and specificities for different diagnostic categories can be found elsewhere [8,15,16]. LUS can additionally be used to guide experimental therapies in complex clinical cases that are not covered by existing guidelines [17].

The pedagogy of acquiring practical LUS skills and the minimum education and training standards required are not yet consistently standardized. Germany, for example, has introduced national training standards and a certification process, but only a few countries have developed standardized LUS education so far [18].

By subanalyzing data from the FASP-ICU trial [19] that compares CXRs and LUS, we can replicate daily clinical ICU practice in a heterogeneous study cohort while minimizing intentional and/or unintentional selection biases. This study, thereby, aims to determine whether LUS in real-world clinical ICU practice (a population of consecutive and unselected patients with different lung diseases) detects more lung abnormalities compared to CXRs.

## 2. Materials and Methods

### 2.1. Patient Enrollment and Institutional Approval

The FASP-ICU (focused assessment of sonographic pathologies in the intensive care unit) study included 111 consecutive ICU patients in two anesthesiology-supervised surgical ICUs with 42 beds. It was conducted at the tertiary university hospital of Goettingen in Germany (1460 beds). The FASP-ICU study was approved by the local ethics committee (ethics proposal Universitaetsmedizin Goettingen 25/6/13, 12 August 2014), and written informed consent was obtained from the participating patients or their legal guardians. The FASP-ICU study was registered with the Germany Clinical Trials Register (DRKS, 7 April 2017) under the identifier DRKS00010428. More detailed information can be found in the original publication of the FASP-ICU trial results [19]. The current study is a subanalysis of the FASP-ICU study results comparing the findings of pulmonary ultrasound examinations with corresponding CXRs.

### 2.2. Ultrasound Techniques and FASP-ICU Study Protocol

In accordance with the FASP-ICU study protocol, patients underwent ocular, vascular, pulmonary, cardiac, and abdominal ultrasound examinations on the day of ICU admission and on days 3, 6, 10, and 15 of their ICU stay. An expert examiner performed all ultrasound examinations. While no international definition of an expert exists, in this case, the physician performing the LUSs was a nationally certified examiner with many years of experience in echocardiography and ultrasonography of different organ systems who also conducted clinical trials in ultrasonography and echocardiography. Images and diagnoses were then verified by a second reviewer who was initially blinded to the diagnoses established by the examiner. In case of a disagreement between the examiner and the reviewer, an agreement was reached by following a previously defined protocol. A radiologist with extensive experience in CXR interpretation in the ICU was blinded to all previous ultrasound diagnoses and reviewed and interpreted all CXRs in accordance with standard radiology guidelines.

A Vivid S5 ultrasound machine from General Electric (GE) with a linear array 6.0–13.00 MHz linear probe, a curved array 1.8–6.0 MHz convex probe, and a phased array adult 1.5–3.6 MHz sector probe was used for the study. All images were digitally stored and immediately analyzed after acquisition.

For pulmonary ultrasound examinations, evidence-based recommendations [20] and techniques that have been previously described were used [21,22]. The examinations were carried out with the combined use of the above-mentioned linear and convex probes by using a manufacturer-defined lung preset that disabled most ultrasound filters, which allowed more reliable detection of pulmonary artifacts. Each examination included the recommended four left and four right chest zones (upper anterior and lateral, lower anterior, and basolateral). When abnormal findings were detected, the examination was expanded to other chest zones and regions as necessary.

Diagnoses were made according to the current evidence-based international recommendations [20], as follows (non-exhaustive listing). Lung consolidation was diagnosed when a tissue-like echotexture/echo-poor region with a loss of lung aeration was present. A pneumothorax was diagnosed when A-lines, the absence of lung sliding, and the presence of a lung point were observed. A diagnosis of interstitial syndrome was based on unilateral or bilateral B-patterns in the absence of other causes of a B-pattern, plus the presence of specific echocardiographic abnormalities. Pleural effusion was diagnosed by detecting an anechoic echotexture with the presence of the quad sign, sinusoid sign, or lung line. The diagnosis of atelectasis was based on lung consolidation plus abolition of lung sliding and the presence of static air bronchograms, whereas for the diagnosis of compression atelectasis, atelectatic lung with partial re-aeration on inspiration in the presence of pleural effusion had to be present.

The procedure for diagnosing interstitial syndrome (abnormal quantity of B-lines, i.e., vertical reverberation abnormalities) and pulmonary edema deviated from current recommendations as described above by taking additional echocardiographic and pulmonary ultrasound findings into account [23]. Details on the echocardiographic evaluation are outlined in the Method section of the FASP-ICU study [19].

### 2.3. Radiological Equipment and Diagnosis

Bedside AP CXRs in the ICU were obtained by employing a Mobilett XP mobile CXR machine (Siemens AG, Munich, Germany) following local protocols. The radiologist reporting the CXRs, who had long-standing experience in ICU thoracic imaging, was blinded to the lung ultrasound examination results, and similarly, the clinician performing the LUS was blinded to the CXR findings. No clinical information was available to the radiologist, whereas the sonographer could theoretically apprehend additional information present at the patient’s bedside. CXRs were defined as corresponding if they were taken within 24 h prior to or after the LUS examination. Retrospective CXR interpretation followed the FASP-ICU LUS categorial assessment based on the categories of consolidation, interstitial syndrome, basal atelectasis, pleural effusion, pneumothorax, compression atelectasis, and hypervolemia.

### 2.4. Data Handling and Statistical Analysis

Data were pseudonymized and digitized by automatic export or by being manually entered into a database. All data were double-checked. Data processing and simple statistical analysis were computed with Microsoft Excel (version 2311 (Build 17029.20068), Redmond, WA, USA). Advanced statistical analysis was performed with SigmaPlot (version 14, Systat Software, San Jose, CA, USA). For the determination of statistical significance, as indicated by a *p*-value below 0.05, LUS and CXR examination results were compared by a paired *t*-test when the Shapiro–Wilk test of normality was passed. All *p*-values are two-tailed. In addition, 95% two-tailed confidence intervals for the difference of means (CI) are reported.

## 3. Results

### 3.1. Patient Characteristics and Number of Examinations

The baseline demographic and clinical characteristics of the patients are displayed in Table 1; more detailed information can be found elsewhere [19]. During the study period, 255 sonographic examinations were conducted on 111 patients during their ICU stay. Up to five serial examinations were performed on each patient [19]. A corresponding CXR was identified for 212 of these examinations (88.14%). Due to subcutaneous emphysema, three examinations could not be compared, resulting in 209 comparable data sets.

### 3.2. Overall Lung Abnormalities

The images were screened for the presence of interstitial syndrome (left, right, bilateral), consolidation (left and/or right apical, medial, basal), basal atelectasis (right and/or left), pleural effusion (right and/or left), compression atelectasis (right and/or left), pneumothorax (right and/or left), and hypervolemia. The number of lung abnormalities and 95% confidence intervals in examinations one to five (E1–E5) are shown in Table 2. Overall, in 209 examinations, LUS detected 1162, and CXR detected 1228 lung abnormalities (*p* = 0.276; CI −0.886 to 0.254; Table 2).

### 3.3. Interstitial Syndrome

Rates of detection of interstitial syndrome by LUS and CXR are shown in Table 3. The incidence of bilateral interstitial syndrome diagnosed by LUS increased during the serial examinations (E1: 10.28%; E2: 19.61%; E3: 26.92%), whereas no trend could be detected by CXR (E1: 5.61%; E2: 3.92%; E3: 7.69%). Agreement rates for LUS and CXR are shown in Table 3. The agreement rate for bilateral interstitial syndrome was highest in E1 (30.8%) and decreased during the serial examinations.

### 3.4. Consolidation

The incidence of consolidation detected by LUS and CXR, as well as agreement rates, are shown in Table 4. There was a trend towards higher agreement rates for later examination time points.

### 3.5. Basal Atelectasis

The incidence of basal atelectasis detected by LUS and CXR is shown in Table 5. The incidence of basal atelectasis as diagnosed by LUS and CXR increased during the serial examinations (E1: LUS left 44.86%, right 30.84%; CXR left 42.99%, right 29.91%; E2: LUS left 64.71%, right 49.02%; CXR left 62.75%, right 35.29%; E3: LUS left 65.38%, right 50%; CXR left 84.62%, right 57.69%; E4: LUS left 50%, right 60%; CXR left 60%, right 30%; E5: LUS left 80%, right 60%; CXR left 60%, right 20%). Agreement rates for LUS and CXR are shown in Table 5. No trend or pattern for the agreement rates between the serial examinations could be observed.

### 3.6. Pleural Effusion

The incidence of pleural effusion detected by LUS and CXR is shown in Table 6. During the serial examinations, the incidence of pleural effusion increased up to E3 (LUS left 57.69%, right 38.46%; CXR left 96.15%, right 57.69%) and, subsequently, decreased. Agreement rates for LUS and CXR are shown in Table 6. No trend or pattern for the agreement rates during the serial examinations could be observed.

### 3.7. Compression Atelectasis

The incidence of compression atelectasis detected by LUS and CXR is shown in Table 7. No trend or pattern could be observed for the incidence in the serial examinations. Agreement rates for LUS and CXR are shown in Table 7. No trend or pattern for the agreement rates during the serial examinations were observed.

### 3.8. Pneumothorax

The incidence of pneumothoraces detected by LUS and CXR is shown in Table 8. Eight out of nine pneumothoraces detected by LUS were found in E1, with the remaining found in E2. All pneumothoraces detected by CXR were found in E1. Agreement rates for LUS and CXR are shown in Table 8. Due to the small number of pneumothoraces, trends or patterns during the serial examinations could not be analyzed.

### 3.9. Hypervolemia

The incidence of hypervolemia detected by LUS and CXR is shown in Table 9. During the serial examinations, the incidence increased up to E3 (LUS 34.62%; CXR 57.69%) and decreased thereafter. Agreement rates for LUS and CXR are shown in Table 9. Agreement rates increased and reached a peak in E3 at 41.18%.

## 4. Discussion

Whereas LUS and CXR found a similar total number of lung abnormalities in the examined categories and during the serial examinations (Table 2), the agreement rates between these two diagnostic tools within all categories were remarkably low (Table 3, Table 4, Table 5, Table 6, Table 7, Table 8 and Table 9). We have deliberately abstained from reporting *p*-values and other statistical indices within the different examined categories and have reported percentages and agreement rates instead. The reason is that apart from computed tomography (CT), there is no overall accepted gold standard diagnostic tool for pulmonary pathologies. Ethical reasons obviously hamper the acquisition of CTs in studies that do not fully replicate clinical practice, such as where CTs may not have been performed or where patients are excluded from a study if appropriate imaging required for the study is not available. As early as 2014, Lichtenstein et al. considered that LUS was the bedside gold standard tool for ICU patients [22], an assertion that is challenged by our study results.

At first glance, the incidence of lung abnormalities in our study appears high. However, it is consistent with previously published data on predominantly anesthetized or sedated ICU patients, which have shown an atelectasis rate of around 90% [24]. As an exception to the various unexpected results of our study, the detection rates for consolidation were higher in the LUS group. This is in accordance with previous evidence, showing a higher diagnostic accuracy for LUS compared with CXR when CT was used as the gold standard [5].

One other study in patients with COVID-19 pneumonia also found a strong disagreement between LUS and CXR; the superiority of LUS was demonstrated by chest CT in selected cases [25]. Agreement rates of roughly 50–70% were found in a limited number of patients after thoracic surgery in a study setting that, like ours, resembled daily clinical routine [26]. In postoperative thoracic surgery patients, an agreement rate of 38% for pleural effusion was reported by Jakobson et al. [27], a rate below even the one reported in this study (Table 6). In the same study, an agreement rate of 72% for the detection of pneumothoraces was found, leading to the conclusion of the authors that LUS could replace CXR in the studied post-thoracic surgery setting [27]. However, this specific study was primarily designed for diagnosing just three categories (pleural effusion, lung consolidation, and pneumothorax in one hemithorax), and therefore, generalizability is limited. Another problem encountered by most studies comparing LUS and CXR is the application of statistical methods when no gold standard method, such as chest CT imaging, is performed. In many cases, clinical diagnosis has been arbitrarily used as a substitute. Neither LUS nor CXR currently represents a true gold standard with high sensitivity and high specificity in the acute setting. Even though CXR is routinely performed, its limitations are well known, especially when the examination is only performed when the patient is in the supine position. As outlined in the section above detailing the patient characteristics, one has to keep in mind that in this study, we report data of critically ill patients of whom up to 55.9% (E1) were mechanically ventilated, a patient population that has so far only seldomly been reported.

In some cases, using either LUS or CXR in isolation might be inappropriate; Vizioli et al. showed that CXR had a high specificity (91%) but low sensitivity (48%) for diagnosing interstitial lung disease, whereas LUS had a high sensitivity (92%) but relatively low specificity (79%), and the authors concluded that CXRs and LUS have different but complementary features [7]. As our study design could not define a gold standard diagnostic tool, we neither state the diagnostic accuracy nor sensitivity or specificity values. However, our data and especially the agreement rates strongly suggest that LUS and CXRs are complementary in clinical practice because each imaging modality detected lung abnormalities that the other modality missed or misdiagnosed.

Although a greater sensitivity of LUS could not be demonstrated in our clinical ICU practice setting, very recently, Volpicelli et al. have raised concern that the superior diagnostic sensitivity of LUS seen in many other studies compared to CXRs might lead to the overdiagnosing and overtreating of lung conditions [28]. Our results partly support this important statement by showing that LUS was presumably diagnosing patients with pulmonary conditions that were not at the time detected by CXR. On the contrary, our treatment algorithms and evidence are often still based on conditions detectable by CXR. To assess the clinical impact of LUS detecting abnormalities with a higher incidence than CXR, well-designed clinical trials that include different imaging modalities as well as clinical features are necessary.

### Limitations of the Study

The present study is a subanalysis of the FASP-ICU trial results, and the primary study design was neither chosen nor adjusted for comparing CXR and LUS. Therefore, CXR and LUS results are compared to each other but not to a gold standard model such as CT. Its results are furthermore impacted by the single-center approach. LUS and CXR analyses were performed within a 24 h time frame, and although this time frame is much shorter than in many other studies comparing these two imaging techniques, one has to bear in mind that critical illness can be very dynamic in nature and that even 24 h can have huge implications on the patient’s clinical status and underlying disease conditions.

Our results could be influenced by the patient population studied. We mainly examined postoperative surgical patients, including after cardiac and thoracic surgical procedures, but evidence around performing LUS is not clearly established in this patient population, and the findings and results may be influenced or altered by a variety of factors, such as patient positioning [8].

The observation of B-lines, suggesting a diagnosis of interstitial lung disease, extravascular lung water, interstitial pneumonia, and other conditions, is not only operator-dependent but is also influenced by a variety of technical parameters, such as imaging frequency [18]. Throughout the study, the same ultrasound machine, with the same probes, preset, and protocol, was used to minimize bias. However, a systemic bias based on technical parameters affecting the quantification of B-lines cannot be completely ruled out.

LUS was performed according to existing guidelines [20] on eight areas of the chest as part of a rapid whole-body ultrasound protocol (FASP-ICU), leaving out most of the posterior areas. A very recent guideline on LUS, unfortunately not available at the time of study execution, now recommends that LUS should be performed on the largest possible area of the chest [18]. It should be noted that the current study followed the guidelines valid at the time of study execution, but of course, a change of practice might have brought different results to an extent that is not describable or quantifiable.

Taking the overall low agreement rates of our study into account, our data indirectly support new recommendations of scanning the largest possible area of the chest in LUS instead of using only a few defined scanning locations.

## 5. Conclusions

In this study, we report evidence that LUS and CXRs in ICU clinical practice do not appear to be equivalent modalities but instead seem to function as complementary diagnostic techniques, consistent with the findings of a review by Marini et al. [3]. Each technique has its unique strengths and limitations, and as shown in our study, the agreement rates can be surprisingly low. As a consequence, using LUS or CXR in isolation may not be appropriate. Instead, this study suggests that a rational approach is to use LUS alongside CXRs and vice versa. Each modality could be superior for different suspected abnormalities in different clinical scenarios and in cases where clinical suspicion is not backed up by LUS or CXR or in patients not responding to treatment.

## Figures and Tables

**Table 1:** Demographics and baseline patient characteristics.

Demographics and Baseline Patient Characteristics				
Gender	male	57.7 (%) 64 (*n*)	female	42.3 (%) 47 (*n*)
Age (years)	mean	68.7 ± 12.9	median	72 (24–90)
Body weight (kg)	mean	79.7 ± 17.3	median	77 (36–150)
Body mass index (kg·m^−2^)	mean	27.0 ± 5.1	median	26.3 (14.1–55.1)
Days in ICU	mean	6.5 ± 8.0	median	3 (1–36)
Catecholamine therapy (at admission)	yes	44.1 (%) 49 (*n*)	no	55.9 (%) 62 (*n*)
Mechanical ventilation (at admission)	yes	55.9 (%) 62 (*n*)	no	44.1 (%) 49 (*n*)
Survival rate	survived	89.2 (%) 99 (*n*)	died	10.8 (%) 12 (*n*)

Continuous variables are expressed as median (range) or mean ± standard deviation, and categorical variables are expressed as percentages.

**Table 2:** Overall lung abnormalities.

Lung Abnormalities					
	LUS	CXR	*n*	*p*	95% CI
Examination 1	501	520	107	0.652	−0.956 to 0.601
Examination 2	322	320	51	0.95	−1.222 to 1.301
Examination 3	183	225	26	0.056	−3.275 to 0.0444
Examination 4	118	138	20	0.284	−2.897 to 0.897
Examination 5	38	25	5	0.15	−1.113 to 5.446
Overall	1162	1228	209	0.276	−0.886 to 0.254

LUS = lung ultrasound; CXR = chest X-ray.

**Table 3:** Incidence and agreement rates for interstitial syndrome.

Interstitial Syndrome	Left Lung	Right Lung	Bilaterally
LUS	13/209 (6.22%)	10/209 (4.78%)	35/209 (16.75%)
CXR	1/209 (0.48%)	2/209 (0.96%)	11/209 (5.26%)
Agreement rate LUS vs. CXR	0% (*n* = 14)	9.1% (*n* = 11)	15% (*n* = 40)

LUS = lung ultrasound; CXR = chest X-ray.

**Table 4:** Incidence and agreement rates for consolidation.

	Left Lung				Right Lung			
Consolidation	Overall	Apical	Medial	Basal	Overall	Apical	Medial	Basal
LUS	148/209 (70.81%)	1/209 (0.48%)	37/209 (17.7%)	148/209 (70.81%)	134/209 (64.11%)	2/209 (0.96%)	20/209 (9.57%)	134/209 (64.11%)
CXR	125/209 (59.81%)	7/209 (3.35%)	41/209 (19.62%)	121/209 (57.89%)	104/209 (49.76%)	20/209 (9.57%)	45/209 (21.53%)	100/209 (47.85%)
Agreement rate LUS vs. CXR	56% (*n* = 175)	14.29%	20%	55.49%	46.01% (*n* = 163)	0%	20.37%	45.34%

LUS = lung ultrasound; CXR = chest X-ray.

**Table 5:** Incidence and agreement rates for basal atelectasis.

Basal Atelectasis	Left Lung	Right Lung
LUS	112/209 (53.59%)	86/209 (41.19%)
CXR	115/209 (55.02%)	72/209 (34.45%)
Agreement rate LUS vs. CXR	49.34% (*n* = 152)	33.90% (*n* = 118)

LUS = lung ultrasound; CXR = chest X-ray.

**Table 6:** Incidence and agreement rates for peural effusion.

Pleural Effusion	Left Lung	Right Lung
LUS	104/209 (49.70%)	79/209 (37.8%)
CXR	142/209 (67.94%)	94/209 (44.98%)
Agreement rate LUS vs. CXR	50% (*n* = 164)	40.65% (*n* = 123)

LUS = lung ultrasound; CXR = chest X-ray.

**Table 7:** Incidence and agreement rates for compression atelectasis.

Compression Atelectasis	Left Lung	Right Lung
LUS	25/209 (11.96%)	21/209 (10.08%)
CXR	95/209 (45.45%)	47/209 (22.49%)
Agreement rate LUS vs. CXR	14.29% (*n* = 105)	19.30% (*n* = 57)

LUS = lung ultrasound; CXR = chest X-ray.

**Table 8:** Incidence and agreement rates for pneumothorax.

Pneumothorax	Left Lung	Right Lung
LUS	4/209 (1.91%)	5/209 (2.39%)
CXR	1/209 (0.48%)	2/209 (0.96%)
Agreement rate LUS vs. CXR	0% (*n* = 5)	0% (*n* = 7)

LUS = lung ultrasound; CXR = chest X-ray.

**Table 9:** Incidence and agreements rates for hypervolemia.

Hypervolemia	
LUS	44/209 (21.05%)
CXR	83/2109 (39.71%)
Agreement rate LUS vs. CXR	20.95% (*n* = 105)

LUS = lung ultrasound; CXR = chest X-ray.

## Data Availability

All data generated or analyzed during this study are included in this published article except the original ultrasound images. These original ultrasound image files (>1 TB of compressed data) are available from the corresponding author upon reasonable request.
